# Effects of Remote Expressive Arts Program in Older Adults with Mild Cognitive Impairment: A Randomized Controlled Trial

**DOI:** 10.3233/JAD-215685

**Published:** 2023-01-17

**Authors:** Yuting Luo, Rong Lin, Yuanjiao Yan, Jiawei Su, Shengmei Lin, Mingping Ma, Hong Li

**Affiliations:** a The School of Nursing, Fujian Medical University, Fuzhou, Fujian, China; b Research Center for Nursing Theory and Practice, Fujian Provincial Hospital, Shengli Clinical Medical College of Fujian Medical University, Fuzhou, Fujian, China; c Department of Radiology, Fujian Provincial Hospital, Fuzhou, Fujian, China

**Keywords:** Aged, art therapy, cognitive dysfunction, magnetic resonance imaging, telemedicine

## Abstract

**Background:**

Mild cognitive impairment (MCI) is a stage of cognitive ability loss with intact activities of daily living and an increased risk for the development of dementia.

**Objective:**

This study evaluated the intervention effect of remote expressive arts program (rEAP) on cognitive function in older adults with MCI and investigated the underlying neurobiological mechanisms.

**Methods:**

We assigned 73 older MCI patients to receive rEAP or health education (HE), who underwent neuropsychological evaluation and resting-state functional magnetic resonance imaging before and after treatment. Neuropsychological scores were analyzed using SPSS software, and regional homogeneity (ReHo) values and seed-based functional connectivity (FC) were analyzed using Matlab software.

**Results:**

The rEAP group showed more significant improvements in cognitive function than the HE group. rEAP affected spontaneous brain activity and brain networks. The ReHo values in the right anterior cingulate/paracingulate cortex and the left dorsolateral superior frontal gyrus significantly increased and decreased, respectively, in the rEAP and HE groups. Further, ReHo value changes were significantly associated with the corresponding neuropsychological test score changes in the rEAP group. Moreover, the rEAP group showed decreased FC between the posterior cingulate cortex and the right middle temporal gyrus and increased FC between the ventromedial prefrontal cortex and left angular gyrus.

**Conclusion:**

The 12-week rEAP improved cognitive function in MCI patients. Additionally, the alterations of spontaneous brain network connections and activity helped improve and maintain cognitive function in MCI patients.

## INTRODUCTION

Mild cognitive impairment (MCI) is an intermediate stage between normal aging and dementia characterized by cognitive impairment according to age and education but with intact activities of daily living [1, 2]. As the annual dementia conversion rate of MCI is 10 times higher than that of normal cognition [3], social isolation during the coronavirus disease (COVID-19) pandemic further accelerated cognitive deterioration [4, 5]. Moreover, there is an urgent need for novel interventions to delay the conversion of MCI to dementia because currently available drugs have limited benefits.

Expressive art program (EAP), a promising nonpharmacologic intervention integrating ≥2 art modalities, including painting, collage, sculpting, storytelling, dance, music, theatre, and others, is an integrated and multi-oriented art treatment [6–8]. EAP is a promising prospect for application in people with cognitive impairment [9]. However, the multiple attributes of EAP lead to more significant heterogeneity among different studies, and research on improving cognitive function by EAP has shown controversial results. Some studies [10, 11] have found that EAP strategies for patients with cognitive impairment do not achieve significant cognitive improvements. These results may be because cognitive measurement indicators selected in the study are not sensitive enough to detect EAP-related benefits in a specific cognitive domain. Conversely, it may also be attributed to the implementation form of EAP. Literature review [12] shows that single-form arts program lacks fundamental mechanisms of cognitive stimulation, such as creativity and reflection. In contrast, EAP includes multi-component, high cognitive complexity, and provides more creative opportunities for participants, which are more likely to bring cognitive benefits [13–15]. To be more specific, the multimodal attributes of EAP provide rich sensory and perceptual stimulation for patients with cognitive impairment [16], promote patients’ self-exploration, and strengthen processing and cooperation among different cognitive domains through the creative expression process, thus improving patients’ cognitive function [13, 17, 18] and mental health [16, 17, 19]. Traditional EAP involves intensive face-to-face classes, which could not be conducted during the COVID-19 pandemic because of public health measures, such as mandatory social distancing [20]. Furthermore, as dementia is a chronic, progressive neurodegenerative disease, long-term sustained interventions are crucial for positive outcomes in chronic diseases [13, 21]. However, long-term, large-scale implementation of face-to-face courses may not be feasible, as they may be inaccessible to patients living in rural areas or patients with limited mobility, and may not be cost-effective. Providing EAP through information and communication technology is an innovative and cost-effective approach for overcoming the limitations of face-to-face interventions, as patients with MCI have equal access and ability to use the Internet as healthy older adults and tend to accept cognitive-improving e-healthcare interventions [22, 23] readily.

Studies have shown the positive effects of EAP on behavioral outcomes in patients with MCI [13, 15]; however, the effects of EAP on neurological function in patients with MCI remain unclear. It has been found that EAP can regulate functional connectivity (FC) in the default mode network (DMN) of adults [24–26]. However, there are limited studies on the effects of EAP on the brain’s functional network in patients with MCI. Resting-state functional magnetic resonance imaging (rs-fMRI) is an excellent tool for detecting regional neural activity and long-distance brain network connections [27]. The regional homogeneity (ReHo) and the region of interesting-based functional connections (ROI-based FC) are two commonly used and reliable rs-fMRI analysis algorithms. ReHo explores the functional connections of local brain regions and has been widely used to explore the pathophysiology of patients with cognitive impairment [28]. Compared with healthy controls, patients with amnestic MCI (aMCI) had significant ReHo changes in brain regions, mainly located in the DMN and executive control network [28]. Some nonpharmaceutical interventions [29] could regulate abnormal ReHo values in patients with MCI, which provides a reference basis for patients with MCI to implement a positive but unique intervention. However, ReHo can only understand the characteristics of local brain activity, while the FC between spatially separated brain regions cannot be detected. The ROI-based FC can make up for this defect. In the context of MCI, the most common resting-state network that affects cognitive dysfunction is the DMN [30]. The medial prefrontal cortex (MPFC) and posterior cingulate cortex (PCC) are the core nodes of the DMN [31] and show the earliest metabolic abnormalities in the precursor stage of Alzheimer’s disease (AD) [32, 33]. The PCC often shows decreased FC in patients with MCI and early AD [34], and the changes in these FCs are also related to the pathological changes causing AD [32]. Therefore, the FCs of these two regions may be used to evaluate the possibility of MCI developing into AD.

In this study, we aimed to evaluate the effects of a remote expressive arts program (rEAP) on cognitive performance in patients with MCI and explore the neuroimaging mechanism of rEAP. In addition to testing the performance of neuropsychological scales in patients with MCI, we tested whether the ReHo value of patients with MCI was affected after an intervention. Furthermore, we picked the PCC and ventromedial PFC (vmPFC) as target regions to analyze whether rEAP affects the FC. We hypothesized that 1) rEAP could enhance the cognitive function of patients with MCI, 2) the change in ReHo value is related to the change in cognitive function of patients with MCI, and 3) the FC of the two regions of the PCC and vmPFC will increase and be related to cognitive changes. However, it is impossible to predict the specific brain regions and patterns of changes in the ReHo value because the ReHo does not need an a priori hypothesis.

## MATERIALS AND METHODS

### Study design

In this single-blind, two-arm, parallel randomized controlled trial, participants were randomly assigned to receive rEAP or health education (HE). The rEAP group completed a 60 min internet-based EAP session twice a week for 12 weeks, administered by the researchers and trained facilitators. The HE group completed HE activities provided by geriatric nurses in Fujian Provincial Hospital twice a week for 12 weeks. Participants were assigned treatments based on computer-generated random numbers sealed by an investigator blinded to the study objectives. Investigators blinded to the group allocation performed assessments at baseline and after the 24 intervention sessions. The Ethics Committee of Fujian Provincial Hospital approved the study (K2020-06-007). Before the trial started, all the participants provided informed consent. This trial has been registered in the Chinese Clinical Trial Registry (ChiCTR2000034465).

### Participant recruitment

**Fig. 1:**
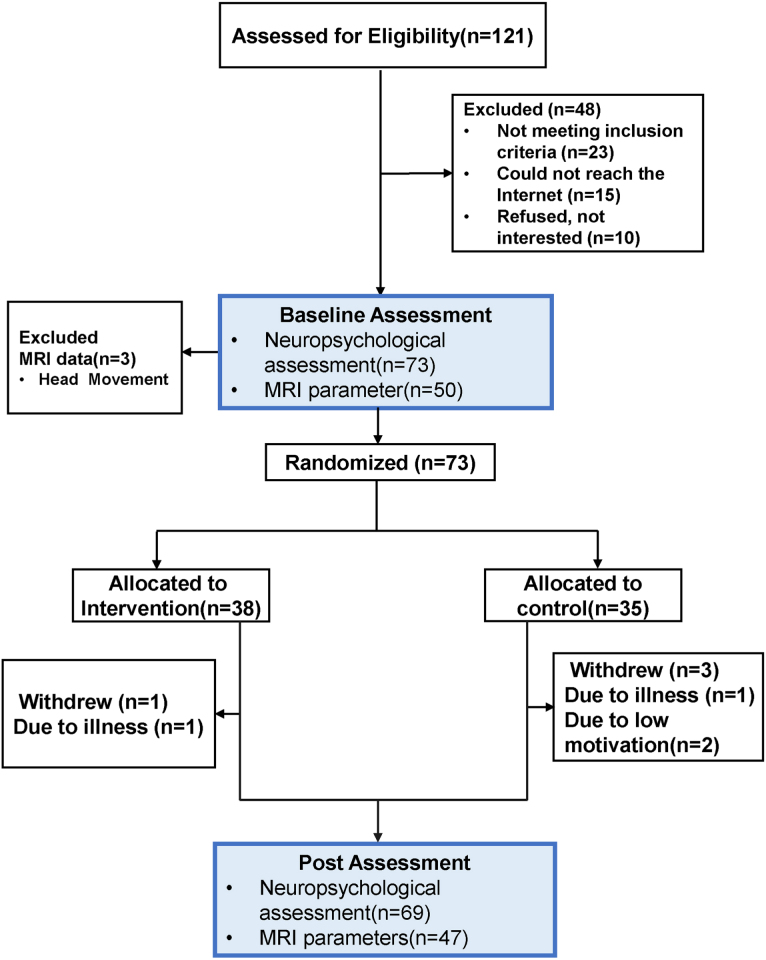
Participant enrollment and inclusion process.

Between July 2020 and August 2021, participants were enrolled from the Memory Clinic of Fujian Provincial Hospital and the Community Health Service Center of Gulou District, Fuzhou, through offline poster posting, online health live streaming, and telephone recruitment (Fig. 1). The sample size was estimated through the “difference test of comparison of two sample means” as follows:

$$n_{1}=n_{2}=2{\left[\frac{\left(u\alpha +u\beta \right)}{\delta /\sigma }\right]}^{2}+\frac{1}{4}u^{2}\alpha$$

Based on a previous study on patients with MCI [13], where the between-group difference in the post-intervention Montreal Cognitive Assessment (MoCA) scores was 1.55, as well as a significance level α= 0.05 (two-tailed) and 1-β= 0.80, we calculated an estimated sample size of 35 participants per group considering a dropout rate of 15%. The inclusion criteria included meeting the Peterson MCI diagnostic criteria [35]; being >60 years old; having normal or corrected vision; ability to access the Internet from an electronic device (e.g., mobile phone, tablet, and computer); being right-handed; having provided informed consent. Additionally, MCI was diagnosed as follows [35]: the presence of an insider-confirmed subjective memory complaint; cognitive impairment according to age and education; ability to perform activities of daily living (ADL) independently; and absence of dementia (Mini-Mental State Examination [MMSE] score ≥17, >20, and >24 for illiterate individuals, individuals educated for 1–6 years, and individuals educated for seven years, respectively).

The exclusion criteria included visual impairment or hearing loss; neurological diseases or other serious chronic diseases; drug or alcohol abuse; participation in another clinical study; and contraindications for MRI, including the presence of metal implants and claustrophobia.

### Interventions

#### rEAP group

**Fig. 2:**
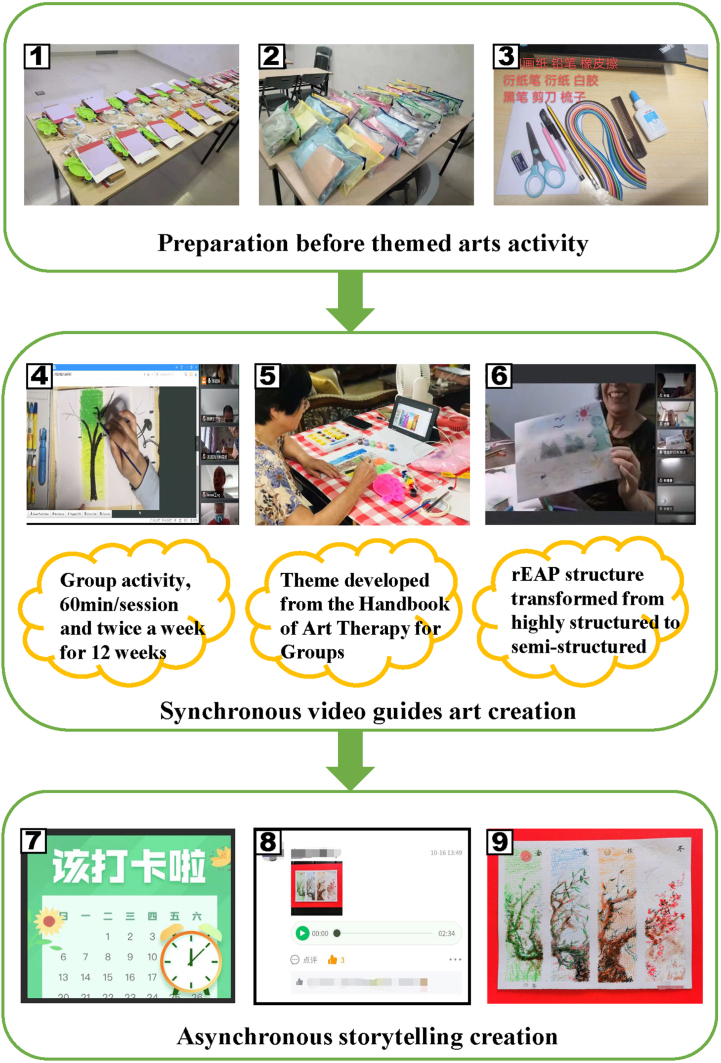
The rEAP procedure. (1)–(3) Art media kits; (4)–(6) Synchronous video guides art creation and interactional feedback; (7)–(9) Recorder of rEAP product.

The rEAP group completed 24 rEAP sessions (Fig. 2, Supplementary Table 1) over 12 weeks using an innovative electronic device (smartphone/tablet/computer). Before treatment, they received art media kits and face-to-face training on remote manipulation; moreover, they were added to a WeChat group chat, where remote activity links and material preparation information were published, followed by check-in management. The rEAP comprised two modules, namely, visual art creation and storytelling. The visual art creation involved synchronous video teaching of art-themed activities. However, each course involved a preset art-themed activity and related media preparation developed based on the Handbook of Art Therapy [36]. We used the Tencent conference “share screen” to share multimedia data and video operation demonstrations. The themed activities gradually changed from structured to semi-structured to make the activities more engaging. More specifically, structured courses were dominated by programmed art education instructions designed to help the older adults with MCI learn to use all types of art materials and organize the designated media creation under a theme. We set up semi-structured courses when participants had a specific art education background. The method implied that the rEAP instructor initiated preset theme-related discussions with the participants to inspire their creative thinking. The participants independently decided the specific creative media types and picture layouts, further increasing the creative space. The subject media was selected according to the Expressive Therapies Continuum theory [37]. In the storytelling module, the participants were asked to create stories based on their visual artworks, with extensive involvement of the “5W1H” principle (who, when, where, what, why, and how). Photos of visual artworks and audio stories were submitted through the WeChat platform.

For intervention fidelity monitoring, the patients were asked to turn on the webcam and microphone during the themed activity to allow interaction with the rEAP instructor to capture the participants’ response to the theme activity and give appropriate guidance when needed. The online art course was set with a password to protect the participants’ privacy. Only the participants in the rEAP group had the link and password to the course. We also told the participants not to disclose the password to others. In addition, as art can arouse deep emotions, if the rEAP instructor perceived sadness, depression, and other adverse emotional reactions from the participants’ voices or videos, we arranged for remote emotional counseling to protect the physical and mental health of the participants. Finally, if the patients encountered complex problems they could not solve, the researchers guided them through WeChat, phone calls, or directly offline.

#### HE group

HE was a 12-week program that included 24 training courses on cognitive health. Specifically, the HE project focused on the determination of participants’ risk and early identification of cognitive impairment. It provided health guidance on maintaining cognitive function around the risk and on protective factors of cognitive impairment, such as dietary advice, methods of physical exercise, and memory strategies in daily life. Participants in the HE group were required to receive 30–60 min of cognitive HE provided by geriatric nurses in the hospital twice a week. The organization mainly included live health lectures and discussion of health tweets in WeChat groups.

### Outcome measures

#### Primary outcome: General cognitive function

The primary outcome measure was general cognitive function determined through the MoCA. The MoCA measures visual-spatial/executive function, naming ability, memory, attention, abstraction, and verbal ability. The total score is 30; a high score represents a better cognitive function. A score of less than 26 is regarded as a cognitive abnormality. If the length of education was less than 12 years, one point was added to the score [38].

#### 2.4.2 Secondary outcomes

Other cognitive measures: The MMSE allows assessment of overall cognitive function [39]; moreover, combining the MoCA and MMSE improves the accuracy of MCI detection [40]. Additionally, we used the auditory verbal learning test (AVLT) to assess memory function [41], in which the abnormal reference values of immediate-term memory were <15 in 50–59 years old, 14 in 60–69 years old, and 13 in 70–79 years old. The critical values of long-term memory abnormalities were 50–59 years old <5 memories, 60–69 years old <4, and 70–79 years old <3. The shape trail test (STT-A and STT-B) was used to assess executive functions [42]. For STT-A, 50–59-year-olds≥the 70 s, 69-year-olds ≥80 s, and 70–79-year-olds ≥100 s, but for STT-B, the abnormal critical value is 50–59-year-olds ≥180 s, 69-year-olds ≥200 s, 70–79-year-olds ≥240 s. Finally, the verbal fluency test (VFT) [43] and the Boston naming test (BNT) were used to assess language proficiency [44]. The VFT required the participants to list as many animals as possible in 1 minute, and the abnormal reference values were defined according to the highest level of education received by the patient. More specifically, abnormal reference values were <11 for junior high school and below, 12 for senior high school, <13 for college and above. The critical point of BNT was <19 for junior middle school, <21 for senior high school, and <22 for college. The standardized symbolic digit modalities test (SDMT) assessed attention and information processing speed [45]. The test requires patients to substitute numbers for abstract symbols using a “key”, and the more correct numbers written within the 90 s were considered better. Functional ability was evaluated using The Lawton Instrumental ADL Scale [46].

### Neuroimaging assessment

(a) Calculation of ReHo value: We used Kendall’s Coefficient concordance to calculate the consistency of activity between a single voxel neuron and surrounding 27 voxel neurons to rapidly map the whole brain’s activity level [47].

(b) Functional connection analysis: We adopted the ROI-based FC analysis method. We selected the bilateral PCC and bilateral vmPFC based on the AAL90 brain atlas [48] and DOS160 brain atlas [49], respectively. Then, the voxel-wise method was used to conduct Pearson’s correlation analysis between the time series of ROIs and the time series of the whole-brain voxel to construct whole-brain FC.

### Data collection

Demographic characteristics (e.g., age, sex, and marital status) were recorded during the first visit. Blinded investigators collected data on neuropsychological and neuroimaging parameters at Fujian Provincial Hospital before and within 2 weeks after the intervention. In addition, the data collection staff received training to standardize neuropsychological measurements and MRI data. To improve test efficiency and reduce unnecessary time delay, we arranged the questionnaire in the following order on the condition that the scale was correctly tested: 1) Complete the overall cognitive assessment first: MoCA, MMSE; 2) Complete the AVLT immediate-term because the AVLT long-term requires a 20-min interval [41]; 3) We prioritized conducting STT and SDMT during the 20-min interval, because these two scales need to be completed by timing and time limit, respectively; 4) after STT and SDMT completion, if there is time remaining, cognitive assessment other than the BNT should be completed to avoid the memory interference of the vocabulary item recognized by BNT in AVLT and affecting the AVLT score; and 5) conduct AVLT long-term and recall-term after 20 min, and then complete the remaining scale. MRI data were collected using a 3.0T magnetic resonance scanner (Siemens Magnetom Prisma, Germany) with a 64-channel head coil for signal reception. We used the following MRI parameters for single-shot echo-planar imaging in rs-fMRI: repetition time (TR) = 2000 ms, echo time (TE) = 30 ms, flip angle (FA) = 90°, field of view (FOV) = 224 mm×224 mm, matrix = 64×64, layer number = 33, voxel = 3.5×3.5×3.5 mm^3^, and layer thickness/layer spacing = 3.5 mm/0.875 mm. We collected data from 240 time points; moreover, the scanning time was 8:06 min. We used the 3D MPRAGE T1WI sequence to obtain whole-brain high-resolution anatomical images for data spatial registration: TR = 2530 ms, TE = 2980 ms, inversion time (TI) = 1100 ms, FA = 7°, FOV = 256 mm×256 mm, matrix = 256×256, layer number = 192, voxel = 1×1×1 mm^3^. Moreover, we used T2-weighted imaging fast spin-echo fluid-attenuated inversion recovery sequence to detect intracranial vascular or space-filling lesions using the following parameters: TR = 5700 ms, TE = 100 ms, FOV = 230 mm×230 mm, matrix = 320×320, layer number = 20, layer thickness/layer spacing = 5 mm/1 mm, number of collections NEX = 1.

Before MRI scanning, the participants were provided individual psychological counseling by the imaging physician and were informed of scanning precautions: no magnetic substances in the body, no clothes except underwear, change into provided examination clothes, and removal of all metal ornaments and removable teeth. The participants were told to lie quietly on the scanning bed with their heads still and eyes closed. However, they were instructed to stay awake and not engage in conscious thinking during the scanning. In addition, to reduce the effect of head movement, the participants were required to wear a noise-resistant earpiece dedicated to magnetic resonance to reduce noise effects. The noise-resistant earpiece filled the space between the head and the coil. During data post-processing, head motion correction was based on a rigid body motion model. It was carried out to eliminate subject data with translational motion higher than 3 mm and rotation greater than 3°. The research team conducted water film and coil performance tests before each formal data collection to ensure that all system indicators complied with fMRI BOLD signal acquisition standards and reduced system noise.

### Statistical analysis

We performed an intention-to-treat analysis. Additionally, missing data were imputed by multiple imputation. Statistical neuropsychological measures were analyzed using IBM SPSS23.0 (IBM, Armonk, New York, USA). Statistical significance tests were two-tailed, with *p* < 0.05. First, baseline characteristics were compared using the Mann–Whitney, Fisher’s, and χ^2^ tests. Second, ANCOVA analyses were conducted to compare neuropsychological indexes between the groups using the respective scores of indexes after intervention as the dependent variables. Age (years), sex, education, and the respective baseline value of neuropsychological indicators were used as covariates.

MATLAB 2013b platform was used for MRI data processing. SPM12 (Statistic Parametric Mapping, https://www.fil.ion.ucl.ac.uk/spm/software/spm12/) and DPARSF (Data processing assistant for resting-state fMRI, https://rfmri.org/DPARSF) software were used for pre-processing the original data, including time-layer correction, head NMO correction, spatial normalization, smoothing, linear drift removal, time filtering, and covariate regression. Regarding the SPM12 software, we used age and sex as covariates, with 2 (inter-group factors: rEAP group and HE group)×2 (intra-group factors: at baseline and after intervention) mixed-factor analysis of variance for the intervention effect on ReHo and FC in patients with MCI. Moreover, we adopted Gaussian random field (GRF) correction. The thresholds of voxel- and cluster-level correction were *p* < 0.001 and *p* < 0.05, respectively. We used the independent sample *t*-test and paired *t*-test to analyze between-group differences in ReHo and FC before and after the intervention. Pearson correlation analysis was used to analyze the correlation between changes in neuropsychological test scores and changes in ReHo and FC.

## RESULTS

Among 121 participants, 73 were considered eligible and randomly categorized into the rEAP group (*n* = 38) and HE group (*n* = 35). MCI subtypes were aMCI and non-amnestic mild cognitive impairment (naMCI) in 37% and 63% of cases, respectively. All participants (N = 73) completed the baseline neuropsychological test. Additionally, 47 of the 73 participants completed two MRI scans, and three patients completed one MRI scan. In the rEAP and HE groups, respectively, one and three participants dropped out of the study (Fig. 1). In the rEAP and HE groups, the total withdrawal rates were 2.63% and 8.57%, respectively.

### Baseline characteristics

Table 1 presents the baseline characteristics of the two groups. Besides, the baseline characteristics of the participants who completed the neuropsychological test and MRI scan were similar (Table 2).

**Table 1:** Baseline characteristics of the study participants

Variable	rEAP group	HE group
	(*n* = 38)	(*n* = 35)
	Median (range)	Median (range)
Age	71.5 (66.0–75.0)	71.0 (65.0–75.0)
**Sex, *n* (%)**
Male	10 (26)	15 (43)
Female	28 (74)	20 (57)
**Education level, *n* (%)**
Primary education	3 (7.9)	3 (8.6)
Junior middle education	7 (18.4)	14 (40.0)
Senior middle education	17 (44.7)	10 (28.6)
>Senior middle education	11 (28.9)	8 (22.9)
**Marital status, *n* (%)**
Single	0 (0.0)	0 (0.0)
Married	29 (76.3)	29 (82.9)
Divorced	1 (2.6)	1 (2.9)
Widowed	8 (21.1)	5 (14.3)
MoCA	23.0 (21.0–25.0)	22.0 (20.0–24.0)
MMSE	27.0 (25.8–28.0)	27.0 (25.0–28.0)
AVLT
Immediate-term	17.0 (14.0–21.3)	15.0 (12.0–18.0)
Long-term	4.0 (3.0–7.0)	3.0 (1.0–5.0)
Recall-term	22.5 (19.8–24.0)	21.0 (18.0–23.0)
VFT	17.0 (15.0–18.3)	15.0 (12.0–19.0)
BNT	22.5 (20.0–24.0)	20.0 (17.0–24.0)
STT
STT-A	58.5 (52.5–74.3)	75.0 (59.0–86.0)
STT-B	157.5 (137.5–190.5)	173.0 (142.0–210.0)
SDMT	33.0 (26.8–37.3)	30.0(24.0–33.0)
**ADL**	20.0 (20.0–20.3)	20.0 (20.0–20.0)

MoCA, Montreal Cognitive Assessment; MMSE, Mini-Mental State Examination; AVLT, Auditory Verbal Learning Test; VFT, Verbal Fluency Test; BNT, Boston Naming Test; STT, Shape Trail Test; SDMT, Symbol Digit Modalities Test; ADL, Activities of Daily Living scale.

**Table 2:** Baseline characteristics of the populations who completed the neuropsychological tests and MRI scans

Variable	Neuropsychological Test	MRI Scan	*Z/* χ^2^	*p* ^*^
	(*n* = 73)	(*n* = 47)
	Median (range)	Median (range)
Age	71.0 (66.0–75.0)	70.0 (65.0–74.0)	–0.783	0.433^a^
**Sex, *n* (%)**			0.573	0.449^b^
Male	25 (34.2)	13 (27.7)
Female	48 (65.8)	34 (72.3)
**Education level, *n* (%)**				0.948^c^
Primary education	6 (8.2)	4 (8.5)
Junior middle education	21 (28.8)	11 (23.4)
Senior middle education	27 (37.0)	19 (40.4)
>Senior middle education	19 (26.0)	13 (27.7)
**Marital status, *n* (%)**			–	0.679^c^
Single	0 (0.0)	0 (0.0)
Married	58 (79.5)	38 (80.9)
Divorced	2 (2.7)	0 (0.0)
Widowed	13 (17.8)	9 (19.1)
MoCA	22.0 (20.0–24.5)	23.0 (21.0–25.0)	0.453	0.651^a^
MMSE	27.0 (25.5–28.0)	27.0 (26.0–28.0)	0.093	0.926^a^
AVLT
Immediate-term	16.0 (13.5–20.0)	17.0 (14.0–21.0)	1.078	0.281^a^
Long-term	4.0 (2.0–6.0)	4.0 (3.0–7.0)	0.990	0.322^a^
Recall-term	22.0 (19.0–23.0)	22.0 (20.0–24.0)	0.850	0.395^a^
VFT	16.0 (14.0–18.5)	17.0(14.0–19.0)	0.756	0.449^a^
BNT	22.0 (19.0–24.0)	22.0 (19.0–24.0)	0.168	0.867^a^
STT
STT-A	62.0 (54.0–80.0)	60.0 (51.0–75.0)	–1.019	0.308^a^
STT-B	163.0 (140.5–197.5)	158.0 (136.0–192.0)	–0.742	0.458^a^
SDMT	32.0 (25.0–35.5)	33.0 (27.0–37.0)	1.355	0.175^a^
ADL	20.0 (20.0–20.0)	20.0 (20.0–21.0)	0.667	0.505^a^

^*^Significance level of α= 0.05 (two-tailed); ^a^Mann–Whitney U test; ^b^chi-square test; ^c^Fisher’s exact probability test; MoCA, Montreal Cognitive Assessment; MMSE, Mini-Mental State Examination; AVLT, Auditory Verbal Learning Test; VFT, Verbal Fluency Test; BNT, Boston Naming Test; STT, Shape Trail Test; SDMT, Symbol Digit Modalities Test; ADL, Activities of Daily Living scale.

### Neuropsychological results

#### Analysis of covariance for between-group comparisons post-treatment

Table 3 shows that there were significant differences in the post-intervention scores of MoCA (*p* = 0.012), MMSE (*p* = 0.035), AVLT long-term (*p* = 0.044), AVLT recall-term (*p* = 0.036), and SDMT (*p* = 0.009) between groups in the mixed MCI group. However, these results did not survive FDR correction.

**Table 3:** Analysis of covariance for between-group comparisons post-treatment

Variables	rEAP		HE		*p* ^*^	*p*
	Before Mean (SD)	After Mean (SD)	Before Mean (SD)	After Mean (SD)		FDR correction
**MoCA**	22.6 (2.9)	24.8 (2.7)	21.6 (3.0)	22.9 (2.7)	**0.012**	0.066
**MMSE**	26.8 (1.9)	27.9 (1.8)	26.7 (1.8)	26.7 (2.5)	**0.035**	0.128
**AVLT**
Immediate-term	17.8 (5.6)	20.1 (6.5)	14.9 (4.9)	16.8 (4.7)	0.314	0.384
Long-term	4.7 (3.0)	6.7 (3.0)	3.6 (2.7)	4.8 (3.0)	**0.044**	0.097
Recall-term	21.4 (2.8)	22.2 (1.7)	20.5 (3.0)	20.7 (2.8)	**0.036**	0.099
**VFT**	17.0 (2.9)	17.3 (4.2)	15.5 (4.1)	14.9 (4.0)	0.154	0.242
**BNT**	22.3 (3.1)	23.2 (3.0)	20.2 (4.2)	20.9 (3.1)	0.069	0.127
**STT**
STT-A	62.6 (14.5)	63.5 (25.7)	73.7 (20.4)	70.5 (19.5)	0.851	0.851
STT-B	163.7 (43.5)	148.3 (58.6)	185.7 (59.5)	163.1 (47.7)	0.841	0.925
**SDMT**	32.6 (8.7)	34.8 (9.6)	29.5 (8.5)	29.1 (8.8)	**0.009**	0.099
**ADL**	20.4 (0.9)	20.1 (0.4)	20.3 (0.7)	20.2 (0.5)	0.248	0.341

^*^Significance level of α= 0.05 (two-tailed); Each analysis of covariance included age, sex, education, and respective baseline value as covariates. MoCA, Montreal Cognitive Assessment; MMSE, Mini-Mental State Examination; AVLT, Auditory Verbal Learning Test; VFT, Verbal Fluency Test; BNT, Boston Naming Test; STT, Shape Trail Test; SDMT, Symbol Digit Modalities Test; ADL, Activities of Daily Living scale.

We further performed exploratory subgroup analysis according to MCI subtypes. In the naMCI subgroup, there were significant differences in the post-intervention scores of MoCA (*p* = 0.010) and MMSE (*p* = 0.044) between groups. Alternatively, in the aMCI subgroup, there were significant differences in the post-intervention score of the AVLT recall term (*p* = 0.028) between groups. These differences were not significant after FDR correction (Supplementary Tables 2 and 3).

### Neuroimaging analysis

Among 50 participants with available imaging data, we excluded three participants (rEAP group, 1; HE group, 2) because of head movement, with 25 and 22 participants in the rEAP and HE groups, respectively, included in the final analysis.

### Effect of both interventions on ReHo

**Fig. 3:**
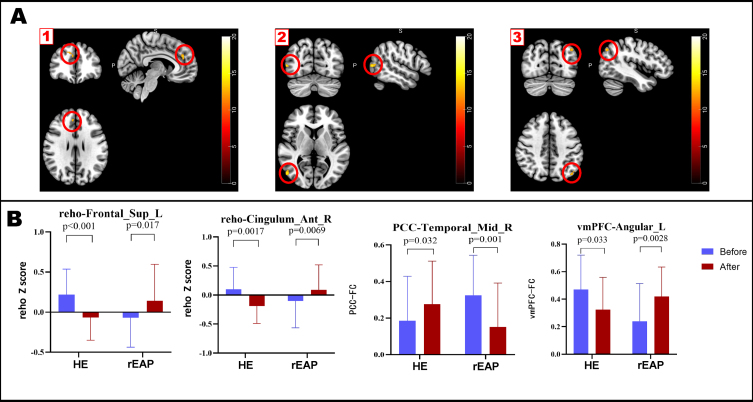
Brain regions with significant interaction and results of the *post-hoc* test. A. 1) ReHo value interaction of significant brain areas; 2) The PCC was used as the ROI showing significant interaction with whole-brain voxel functional connections; 3) The vmPFC was used as the ROI showing significant interaction with whole-brain voxel functional connections. B. The *post-hoc* examination of brain regions showing significant interaction. Color bars are presented with F values, and the error bar represents the standard deviation. ReHo, regional homogeneity; Cingulum_Ant_R, right anterior cingulate/paracingulate cortex; HE, health education; rEAP: remote expression arts program; Frontal_Sup_L, left dorsolateral superior frontal gyrus; PCC, posterior cingulate cortex; Temporal_Mid_R, right middle temporal gyrus; Angular_L, left angular gyrus.

**Fig. 4:**
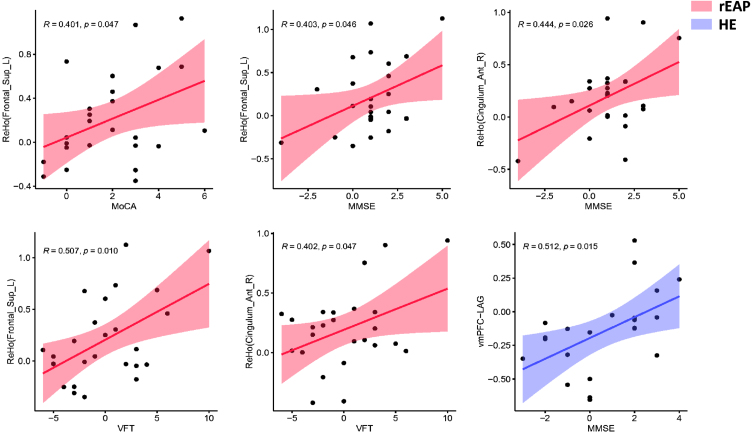
Scatter plot of correlations between altered neuroimaging indexes and neuropsychological performance. The changes in ReHo values in the right anterior cingulate/paracingulate gyrus and the left dorsolateral superior frontal gyrus were positively correlated with changes (post-pre) in MoCA, MMSE, and VFT scores in the rEAP group. The FC alterations between the vmPFC and left angular gyrus were positively correlated with changes in MMSE scores in the HE group. Colored areas represent the 95% confidence interval.

There was no significant between-group difference in the baseline ReHo values. Interaction analysis (Fig. 3, Table 4) revealed significant between-group differences in the ReHo values in the right anterior cingulate/paracingulate cortex (ACC) and the left dorsolateral superior frontal gyrus (dl-SFG) (GRF-corrected, voxel *p* < 0.001, cluster *p* < 0.05). Compared with the HE group, the rEAP group showed a post-intervention increase in the ReHo values in the right ACC (*p* < 0.05) and the left dl-SFG (*p* < 0.05). The changes in ReHo values in the right ACC and the left dl-SFG were positively correlated with changes in MoCA, MMSE, and VFT scores in the rEAP group. In contrast, no correlations were found between alterations in ReHo values and changes in neuropsychological test scores in the HE group (Fig. 4, Table 5).

**Table 4:** Brain regions with significant interaction

Brain regions	Template	Voxels	MNI coordinates			F-value
			X	Y	Z
Cingulum_Ant_R	AAL	14	6	42	24	16.49
Frontal_Sup_L	AAL	22	–21	15	48	17.49
Temporal_Mid_R	AAL	12	54	–72	6	18.20
Angular_L	AAL	6	–45	–75	42	15.80

Cingulum_Ant_R, right anterior cingulate/paracingulate cortex; Frontal_Sup_L, left dorsolateral superior frontal gyrus; Temporal_Mid_R, right middle temporal gyrus; Angular_L, left angular gyrus.

**Table 5:** Correlations between altered neuroimaging indexes and changes in neuropsychological test scores

Indicators	Group	ReHo (Frontal_Sup_L)		ReHo (Cingulum_Ant_R)		PCC-RMTG		vmPFC-LAG	
		*r*	*p*	*r*	*p*	*r*	*p*	*r*	*p*
MoCA	rEAP	**0.401**	**0.047**	0.315	0.125	–0.313	0.128	–0.179	0.393
	HE	–0.033	0.884	0.340	0.122	–0.145	0.520	0.134	0.552
MMSE	rEAP	**0.403**	**0.046**	**0.444**	**0.026**	–0.259	0.212	–0.215	0.303
	HE	0.138	0.539	–0.029	0.897	0.222	0.320	**0.512**	**0.015**
AVLT
Immediate-term	rEAP	0.019	0.927	–0.215	0.303	0.073	0.729	0.151	0.472
	HE	0.191	0.396	0.124	0.581	–0.141	0.531	–0.042	0.852
Long-term	rEAP	0.124	0.556	–0.023	0.915	–0.251	0.225	–0.279	0.177
	HE	0.306	0.166	–0.001	0.995	–0.081	0.720	0.165	0.462
Recall-term	rEAP	0.070	0.739	–0.010	0.964	0.138	0.509	0.227	0.274
	HE	–0.048	0.831	–0.279	0.208	–0.098	0.665	0.055	0.807
VFT	rEAP	**0.507**	**0.010**	**0.402**	**0.047**	–0.226	0.277	–0.147	0.482
	HE	0.238	0.286	–0.252	0.258	0.027	0.905	–0.412	0.057
BNT	rEAP	–0.181	0.387	0.239	0.250	–0.221	0.288	–0.224	0.282
	HE	0.402	0.064	–0.025	0.913	–0.094	0.678	0.303	0.170
STT
STT-A	rEAP	–0.374	0.065	–0.044	0.835	–0.121	0.565	–0.108	0.608
	HE	–0.061	0.788	–0.173	0.441	–0.089	0.693	0.241	0.280
STT-B	rEAP	–0.200	0.338	–0.196	0.348	0.004	0.986	–0.160	0.446
	HE	0.362	0.098	0.336	0.126	–0.210	0.347	0.240	0.282
SDMT	rEAP	0.140	0.506	0.187	0.370	–0.194	0.352	–0.034	0.872
	HE	–0.083	0.715	0.168	0.456	–0.060	0.790	0.033	0.883

MoCA, Montreal Cognitive Assessment; MMSE, Mini-Mental State Examination; AVLT, Auditory Verbal Learning Test; VFT, Verbal Fluency Test; BNT, Boston Naming Test; STT, Shape Trail Test; SDMT, Symbol Digit Modalities Test; ADL, Activities of Daily Living Scale; Cingulum_Ant_R, right anterior cingulate/paracingulate cortex; Frontal_Sup_L, left dorsolateral superior frontal gyrus; RMTG, right middle temporal gyrus; LAG, left angular gyrus.

Furthermore, in the naMCI subgroup, changes in the ReHo value in the left dl-SFG were positively and negatively correlated with changes in VFT and STT-A scores in the rEAP group, respectively. However, the changes in the ReHo value of the left dl-SFG and the STT-A score in the HE group were negatively correlated. In the aMCI subgroup, there was a positive correlation between the changes in the ReHo value and MMSE score in the rEAP group. However, the changes of the ReHo value in the left dl-SFG were positively and negatively correlated with the changes of MMSE and SDMT scores in the HE group (Supplementary Tables 4 and 5).

### Effects of both interventions on functional connectivity in brain regions

There were no significant between-group differences in baseline FC between the PCC and whole-brain voxels. Interaction effect analysis (Fig. 3, Table 4) revealed significant between-group differences in the changes in the FC between the PCC and right middle temporal gyrus (RMTG) (GRF correction, Voxel *p* < 0.001, Cluster *p* < 0.05). Compared with the HE group, the rEAP group showed decreased FC between the PCC and RMTG. There was no significant between-group difference in FC between the baseline vmPFC and whole-brain voxels. Interaction effect analysis (Fig. 3, Table 4) revealed significant between-group differences in FC changes between the vmPFC and left angular gyrus (LAG) (GRF correction, Voxel *p* < 0.001, Cluster *p* < 0.05). Moreover, the rEAP group showed a post-intervention increase in the FC between the vmPFC and LAG, which weakened in the HE group. No correlation of FC changes with neuropsychological test scores was identified in the rEAP group. In contrast, the FC alterations between the vmPFC and LAG were positively correlated with changes in MMSE scores in the HE group (Fig. 4, Table 5).

In addition, exploratory subgroup analysis revealed that changes in FC in PCC-RMTG after rEAP treatment in the naMCI subgroup were negatively correlated with changes in AVLT long term scores. In contrast, FC changes between vmPFC and LAG were negatively correlated with changes in VFT scores in the HE group. In the aMCI subgroup, FC changes in vmPFC-LAG were negatively correlated with changes in STT-B scores in the rEAP group but positively correlated with changes in BNT scores in the HE group (Supplementary Tables 4 and 5).

## DISCUSSION

This study is the first to implement rEAP in patients with MCI and confirm the effect of rEAP on functional brain networks in patients with MCI using resting-state fMRI. We initially observed that the rEAP group positively affected cognitive function. Furthermore, the increase in ReHo values in the brain regions related to the executive control network in the rEAP group is related to improving cognitive function. Therefore, it implies that rEAP can effectively improve cognitive function in patients with MCI; moreover, it can maintain cognition by improving the patterns of brain activity in patients with MCI. Additionally, this study demonstrated the feasibility of using internet technology to conduct proactive e-health interventions in patients with MCI.

### rEAP is associated with changes in cognitive function and neuroimaging

After rEAP treatment, the increased ReHo values in the right ACC and left dl-SFG in patients with MCI were related to improving overall cognitive function and verbal ability. The frontal lobe plays a vital role in cognitive functions such as memory and executive functions. The superior frontal gyrus (SFG) is anatomically connected to the middle frontal gyrus through arcuate fibers in the dorsolateral area. The SFG also has positive functional connections with the lateral prefrontal lobe and the posterior cingulate/precuneus. These findings indicate that the dorsolateral SFG may be one of the nodes of the executive cognitive network involved in initiating and regulating cognitive control [50]. The ACC is responsible for integrating multiple information sources such as emotion, sensation, motor, and cognition and plays a vital role in reinforcement learning, conflict control, and error monitoring. It is a brain region highly related to cognition and emotion management [51, 52]. Our findings show that rEAP may improve cognitive function by increasing the consistency of local activity in cognitively related executive control brain regions.

The rEAP group accepted the use of art media to complete different art training themes. The participants need comprehensive information such as through vision, hearing, working memory and short-term memory retention, media operation skills, goal-oriented planning steps, and idea visualization. They also need continuous self-monitoring and iteration between different ideas to meet art themes. These processes stimulate executive control networks involved in creativity, planning, decision-making, and cognitive control [24]. They may translate stimuli in multiple cognitive domains, such as visuospatial, attention, memory, executive function, and speech, into gains in overall cognitive function. Verbal fluency tasks typically require various executive control processes (such as focusing on task information, updating materials, and suppressing extraneous responses) [53]. Although the SFG and ACC are not usually included in language production patterns, previous studies have shown that they are involved in semantic interference processes [54]. Activation in the SFG is interpreted as an attempt to suppress control and extraneous information [55], and the ACC is thought to be used to monitor the existence of competition [56]. Therefore, we speculate that the positive association between rEAP and the verbal function of patients with MCI may be recruiting brain areas of the executive control network to monitor the semantic information that has nothing to do with the output of the target words. At the same time, it reduces the interference of irrelevant semantics to output the target semantic content.

Although the benefits of rEAP did not survive FDR correction, we observed the potential of rEAP to improve memory and attention in the older adults with MCI. We did not expect that rEAP would affect memory function, and we speculate that the main reason for this phenomenon is the heterogeneity of MCI. The older patients with MCI included in this study were patients with mixed MCI. In contrast, the patients with naMCI in the rEAP group were widely distributed, which may have caused the difference in memory performance between the two groups at baseline. Although we adjusted for the effect of baseline memory performance, the heterogeneity of MCI may be responsible for the fact that the rEAP group was more likely to obtain memory benefits than the HE group. For the attention benefit, we think it is associated with how the rEAP is delivered. The rEAP requires participants to listen carefully to the guide and try and practice art creation repeatedly. In this process, participants need to be immersed in the flow associated with increased attention and continuous participation. Participants often need to integrate a wide range of information, including information detection, identification, discrimination, and attention switching between multi-tasking, to promote the visualization of artistic creation, and such training will naturally translate into the benefits in attention tests [12], such as the SDMT. However, improvements in both cognitive domains were not found to be associated with changes in neuroimaging.

Given that most of the patients in this study were patients with naMCI, naMCI can be impaired in any non-memory cognitive domain, which creates heterogeneity in the naMCI subpopulation. However, memory and attention performance are closely related to executive function in patients with naMCI, often assisted by distributed neural networks [57, 58]. Thus, the differences between neuropsychological and neuroimaging findings may be attributed to the heterogeneous and diffuse pattern of neurodegeneration found in naMCI.

### Subgroup analysis based on MCI subtype

We also tried to perform subgroup analysis based on the MCI subtype. There were significant differences in AVLT recall term in the aMCI subgroup between the two groups after the intervention. The changes in ReHo values in dl-SFG and ACC were positively correlated with improving overall cognitive function, and FC changes in vmPFC-LAG was negatively correlated with the STT-B score. The vmPFC is the core node of the former default mode subnetwork. Studies have found that FC in anterior DMN of patients with aMCI increases [59], which is considered a compensatory increase in cognitive function to maintain task performance. Therefore, the increased FC of vmPFC-LAG in the rEAP group may be a compensatory effect and synergistic with the high ReHo index of the executive control brain region to improve the executive function of patients with aMCI. It is worth noting that the correlation between the change in the ReHo index of the right ACC and the improvement of the cognitive domain was observed only in the rEAP group, which may indicate that the mode of action of rEAP is different from that of HE. As the core area of the salience network, ACC participates in the integration and coordination of information to guide behavior and plays a crucial role in the conflict monitoring process of attention tasks [60]. Studies have found that there are inhibitory control deficits in patients with aMCI [61], and it is often observed that the ReHo value of ACC in patients with aMCI is lower than that in healthy older people, indicating that the local consistency of ACC is significantly decreased in patients with aMCI [62]. AVLT recognition is an episodic memory task requiring cognitive control, which requires participants to identify the learned target words among the interference words. Although the neuroimaging indexes in the rEAP group were not correlated with the changes in AVLT recognition, we hypothesized that the enhanced local coherence of ACC in the rEAP group might improve the cognitive control and conflict monitoring of episodic memory tasks by improving the inhibitory control ability of patients with aMCI. Therefore, it showed the difference in AVLT recall term between the two groups in the aMCI subgroup.

In the naMCI group, we found significant differences in global cognitive function between the two groups after the intervention. The change of ReHo value in the rEAP group was associated with improving verbal and executive domains. In contrast, the improvement of delayed recall was negatively correlated with the change of FC in PCC. Previous studies have found that impaired delayed recall may be because of AD-specific deficits in learning and storage processes and inefficient encoding or retrieval strategies caused by poor execution in patients with naMCI, which may lead to decreased memory performance [63].

Interestingly, we did not observe the association between the increase of ReHo value in the executive brain region and memory improvement in the rEAP group. However, we observed that the decreased FC value of PCC-RMTG was negatively correlated with AVLT long term score, similar to the results of previous studies [64], indicating that increased PCC connectivity in patients with naMCI may predict poor episodic memory retrieval. PCC is involved in extracting episodic memory [65] and is also the region likely to produce AD-related pathological protein deposition [66]. Therefore, it is speculated that rEAP may reduce the neural interference in episodic memory-related brain regions, reduce the neural activation required to perform memory tasks, generate more effective neural processing, and improve memory function. While there were significant differences between groups in overall cognitive function among naMCI participants, no differences were found in cognitive subdomains. We speculate that the rEAP may increase the execution control network coordination and enhance neural processing efficiency in episodic memory-related regions to produce tiny improvements in cognitive subdomains. Therefore, the accumulations of minor alterations in cognitive subdomains produced a marginal effect, and finally the difference in overall cognitive function between the rEAP and HE groups was facilitated.

### The association between HE and cognitive changes

Notably, cognitive benefits were also observed in the HE group. Regarding the cognitive benefits of HE, we believe this may be attributed to the higher brain plasticity of patients with MCI [67, 68]. Cognitive stimulation can also be exerted with educational activities [69]. Furthermore, HE activities are also cognitive tasks, such as paying attention to the health course and thinking about some discussion questions, which activate the brains of the HE group, as Masika et al. [70] reported. In addition, by having access to health knowledge, the HE group may change their health concepts and consciously improve or maintain their cognitive health behavior, eventually improving their overall cognitive function. Our exploratory subgroup analysis also found that the improvement of cognitive domains in HE group was not only related to the FC changes of vmPFC but also related to the ReHo value of dl-SFG. It is worth noting that the development trend of neuroimaging indexes in the HE group seems to be opposite to that in the rEAP group, and the ReHo value of dl-SFG and FC of vmPFC-LAG decreased after the intervention. We hypothesized that the HE group may improve cognition not by enhancing cognition and neural reserve of patients with MCI but by increasing the ability to activate neural tissues to reduce neural workload during cognitive tasks, increasing the efficiency of brain regions, and improving cognitive function. However, this explanation is speculative and should be viewed with caution because we did not include healthy controls and the number of voxels detected in the LAG was small, indicating that the rsFC between the vmPFC and the LAG may be minimal.

This study has some limitations. First, given that we performed a single-center study with relatively small sample size, the documented findings should have been confirmed in a larger sample. Second, the effect of rEAP may have been highlighted in that the average level of neuropsychological tests in the control group at baseline in this study was poorer. In addition, we did not quantify the amount of time the participants spent in contact with the researcher in each arm; therefore, we could not assess the impact of this potentially important variable. Due to the limited time and budget, this study did not include healthy controls, so conclusions related to functional connectivity should be carefully examined. Besides, the number of voxels detected in this study was small. However, this may be because brain function change is a relatively slow process, and the intervention cycle in this study was only 12 weeks. Short-term artistic interventions may not be enough to make a significant difference in brain function. In addition, the fMRI data loss rate was also significant, with one-third of the patients in the study not participating in fMRI measurements, which may have limited the study’s ability to detect significant therapeutic effects of rEAP after the intervention. The main reason for this attrition is that many patients refuse to participate in fMRI scanning because they worry about physical discomfort caused by scanning. However, some patients have relative contraindications to scanning. Future studies should expand the sample size to retain as many participants qualified for fMRI as possible. Finally, in this study, the neuropsychological assessment was only recorded offline, and future studies could be designed so that both assessment and intervention could be completed remotely. Extending the scope of the study and increasing the follow-up time would also better highlight the duration of long-term effects caused by the application of treatment.

Conclusively, our study shows that older patients with MCI can benefit from rEAPs and yield improvements in cognitive function, ReHo, and FC activity in the DMN brain regions. This study supports the feasibility and benefits of rEAP for cognitive maintenance, which is especially important for individuals with limited access to treatment because of geographical distance, transportation barriers, or lack of local services. Additionally, the COVID-19 pandemic has emphasized the importance of remote cognitive stimulation and social support programs to ensure long-term continuous cognitive management of patients with cognitive impairments and delay cognitive decline.

## Supplementary Material

Supplementary Material
