# Supplementary material for: Effects of Remote Expressive Arts Program in Older Adults with Mild Cognitive Impairment: A Randomized Controlled Trial
Source: J Alzheimers Dis. 2023 Jan 17;91(2):815–31. doi: 10.3233/JAD-215685 (PMC9912742; doi:10.3233/JAD-215685)
Supplement: Supplementary Material [file jad-91-jad215685-s001.pdf]

# Supplementary Material

## Effects of Remote Expressive Arts Program in Older Adults with Mild Cognitive Impairment: A Randomized Controlled Trial

**Supplementary Table 1.** Sessions of remote expressive arts program

| Session name | Theme | Structure | Main materials |
|---|---|---|---|
| Session 1: Wild Scenery | Historical nostalgia | Structured | In16k paper, pastels, color pencil, scissors, eraser |
| Session 2: Sunflowers in summer | Realistic orientation | Structured | In16k paper, pastels, pencil, scissors, eraser |
| Session 3: Paper-rolling card | General themes | Structured | In16k paper, paper roller, white latex, pen, paper-rolling pen |
| Session 4: Autumn Chrysanthemum | Realistic orientation | Structured | In16k paper, paper roller, white latex, pen, paper-rolling pen |
| Session 5: Botanic Kingdom | General themes | Structured | Clay, paper disc, Clay sculpting knife |
| Session 6: Underwater world | General themes | Structured | Clay, paper disc, Clay sculpting knife |
| Session 7: Street Corner | Realistic orientation | Structured | In16k paper, color pencil |
| Session 8: Mid-Autumn Festival | Historical nostalgia | Structured | In16k black thick paper, In16k paper, watercolor, brush, glue, scissors |
| Session 9: National Day | Historical nostalgia | Structured | In16k colored thick paper, glue, scissors, paper-rolling pen, paper roller |
| Session 10: Urban Night | Realistic orientation | Structured | In16k paper, color pencil |
| Session 11: Jiangnan Impression | Realistic orientation | Structured | paper disc, watercolor, brush |
| Session 12: Reflection in Water | General themes | Structured | In16k paper, watercolor, brush |
| Session 13: Four seasons | General themes | Structured | In16k paper, Oil pastels, watercolor, brush |
| Session 14: Meaningful seasons | Historical nostalgia | Semi-structured | In16k paper, independent selection of other media |
| Session 15: Fairy World | Guided imagery | Semi-structured | Clay, paper disc, Clay sculpting knife |
| Session 16: Imaginary Creature | Guided imagery | Semi-structured | In16k paper, watercolor, brush |
| Session 17: Wonderland | Guided imagery | Semi-structured | In16k paper, Oil pastels |
| Session 18: Microscopic View | Guided imagery | Semi-structured | In16k paper, independent selection of other media |

| | | | |
|---|---|---|---|
| Session 19: The Moral of the Fish | Historical nostalgia | Semi-structured | In16k paper, paper roller, white latex, paper-rolling pen, independent selection of other media |
| Session 20: Dress Production | General themes | Semi-structured | In16k paper, paper roller, white latex, paper-rolling pen, independent selection of other media |
| Session 21: Primitive Tribe | Historical nostalgia | Semi-structured | In16k paper, pastels, color pencil |
| Session 22: Word Association | Guided imagery | Semi-structured | In16k paper, independent selection of other media |
| Session 23: Random Selection | Guided imagery | Semi-structured | In16k paper, independent selection of other media |
| Session 24: Window Scenery | Realistic orientation | Semi-structured | In16k paper, independent selection of other media |

**Supplementary Table 2.** Analysis of covariance for between-group comparisons posttreatment in naMCI subgroup

| Variables | rEAP(n=27) | | HE(n=19) | | | |
|---|---|---|---|---|---|---|
| | Before Mean (SD) | After Mean (SD) | Before Mean (SD) | After Mean (SD) | *p** | *p* FDR correction |
| **MoCA** | 23.1 (2.4) | 25.5 (2.1) | 22.8 (2.2) | 23.6 (2.2) | **0.010** | 0.110 |
| **MMSE** | 27.3 (1.6) | 28.3 (1.5) | 27.3 (1.5) | 27.3 (2.5) | **0.044** | 0.242 |
| **AVLT** | | | | | | |
| Immediate-term | 19.7 (5.3) | 22.0 (5.5) | 17.5 (4.2) | 18.8 (5.1) | 0.216 | 0.339 |
| Long-term | 5.9 (2.5) | 7.8 (2.5) | 5.5 (2.1) | 6.1 (2.6) | 0.079 | 0.290 |
| Recall-term | 22.3 (2.2) | 22.6 (1.6) | 22.3 (1.5) | 22.1 (1.7) | 0.516 | 0.568 |
| **VFT** | 17.6 (2.2) | 18.0 (3.4) | 16.6 (3.5) | 16.2 (4.5) | 0.394 | 0.542 |
| **BNT** | 22.2 (2.9) | 23.4 (2.7) | 21.3 (4.0) | 21.4 (3.4) | 0.079 | 0.217 |
| **STT** | | | | | | |
| STT-A | 63.2 (14.1) | 64.3 (26.1) | 65.9 (14.8) | 60.2 (15.5) | 0.489 | 0.598 |
| STT-B | 160.9 (38.7) | 144.1 (59.3) | 159.1 (32.5) | 147.2 (43.2) | 0.869 | 0.869 |
| **SDMT** | 33.4 (7.5) | 35.9 (9.3) | 33.7 (7.0) | 33.5 (8.0) | 0.099 | 0.218 |
| **ADL** | 20.3 (0.5) | 20.1 (0.4) | 20.2 (0.5) | 20.3 (0.7) | 0.192 | 0.352 |

*Significance level of $\alpha = 0.05$ (two-tailed); Each analysis of covariance included age, sex, education, and respective baseline value as covariates.

MoCA, Montreal Cognitive Assessment; MMSE, Mini-Mental State Examination; AVLT, Auditory Verbal Learning Test; VFT, Verbal Fluency Test; BNT, Boston Naming Test; STT, Shape Trail Test; SDMT, Symbol Digit Modalities Test; ADL, Activities of Daily Living scale

**Supplementary Table 3.** Analysis of covariance for between-group comparisons posttreatment in aMCI subgroup

| Variables | rEAP(n=11) | | HE(n=16) | | | |
|---|---|---|---|---|---|---|
| | **Before Mean (SD)** | **After Mean (SD)** | **Before Mean (SD)** | **After Mean (SD)** | ***p**** | ***p* FDR correction** |
| **MoCA** | 21.4 (3.5) | 23.3 (3.6) | 20.2 (3.3) | 21.9 (3.0) | 0.469 | >0.999 |
| **MMSE** | 25.5 (2.2) | 26.7 (2.0) | 25.9 (1.8) | 26.0 (2.4) | 0.558 | >0.999 |
| **AVLT** | | | | | | |
| Immediate-term | 13.4 (3.6) | 15.4 (6.4) | 11.9 (3.8) | 14.4 (2.9) | 0.850 | 0.935 |
| Long-term | 1.6 (1.4) | 4.1 (2.7) | 1.3 (1.1) | 3.3 (2.9) | 0.839 | >0.999 |
| Recall-term | 19.4 (3.1) | 21.4 (1.9) | 18.3 (2.8) | 19.1 (3.1) | **0.028** | 0.308 |
| **VFT** | 15.5 (3.8) | 15.5 (5.4) | 14.3 (4.4) | 13.3 (2.7) | 0.394 | >0.999 |
| **BNT** | 22.5 (3.5) | 22.7 (3.6) | 18.8 (4.2) | 20.3 (2.7) | 0.581 | 0.913 |
| **STT** | | | | | | |
| STT-A | 61.2 (16.2) | 61.5 (26.0) | 82.9 (22.7) | 82.7 (16.6) | 0.092 | 0.337 |
| STT-B | 170.7 (55.0) | 158.5 (58.4) | 217.2 (69.3) | 181.9 (47.1) | 0.878 | 0.878 |
| **SDMT** | 30.5 (11.3) | 32.2 (10.3) | 24.4 (7.4) | 23.9 (6.7) | 0.076 | 0.418 |
| **ADL** | 20.7 (1.3) | 20.1 (0.3) | 20.4 (0.8) | 20.1 (0.3) | 0.847 | >0.999 |

*Significance level of $\alpha = 0.05$ (two-tailed); Each analysis of covariance included age, sex, education, and respective baseline value as covariates.

MoCA, Montreal Cognitive Assessment; MMSE, Mini-Mental State Examination; AVLT, Auditory Verbal Learning Test; VFT, Verbal Fluency Test; BNT, Boston Naming Test; STT, Shape Trail Test; SDMT, Symbol Digit Modalities Test; ADL, Activities of Daily Living scale

**Supplementary Table 4.** Correlations between altered neuroimaging indexes and changes in neuropsychological test scores among naMCI subgroup

| **Indicators** | **Group** | **ReHo (Frontal_Sup_L)** | | **ReHo (Cingulum_Ant_R)** | | **PCC-RMTG** | | **vmPFC-LAG** | |
|---|---|---|---|---|---|---|---|---|---|
| | | **r** | ***p*** | **r** | ***p*** | **r** | ***p*** | **r** | ***p*** |
| MoCA | rEAP | 0.254 | 0.293 | 0.138 | 0.574 | -0.151 | 0.538 | -0.107 | 0.663 |
| | HE | -0.107 | 0.717 | 0.255 | 0.380 | -0.086 | 0.769 | 0.255 | 0.379 |
| MMSE | rEAP | 0.042 | 0.865 | 0.151 | 0.536 | -0.175 | 0.473 | -0.266 | 0.270 |
| | HE | -0.137 | 0.641 | -0.150 | 0.609 | 0.303 | 0.293 | 0.432 | 0.123 |
| AVLT | | | | | | | | | |
| Immediate-term | rEAP | 0.076 | 0.756 | -0.176 | 0.472 | -0.075 | 0.760 | 0.128 | 0.601 |
| | HE | 0.091 | 0.757 | 0.213 | 0.464 | -0.209 | 0.474 | -0.356 | 0.211 |
| Long-term | rEAP | 0.416 | 0.077 | 0.083 | 0.735 | **-0.494** | **0.031** | -0.258 | 0.287 |
| | HE | 0.427 | 0.128 | 0.029 | 0.922 | -0.222 | 0.445 | -0.034 | 0.909 |
| Recall-term | rEAP | -0.032 | 0.898 | 0.105 | 0.668 | 0.026 | 0.915 | 0.192 | 0.431 |
| | HE | 0.007 | 0.981 | -0.372 | 0.191 | -0.399 | 0.158 | -0.264 | 0.362 |
| VFT | rEAP | **0.532** | **0.019** | 0.356 | 0.135 | -0.329 | 0.169 | -0.266 | 0.271 |
| | HE | 0.043 | 0.885 | -0.504 | 0.066 | 0.224 | 0.442 | **-0.552** | **0.040** |
| BNT | rEAP | -0.075 | 0.760 | 0.311 | 0.195 | -0.448 | 0.054 | -0.314 | 0.190 |
| | HE | 0.411 | 0.144 | 0.013 | 0.965 | -0.101 | 0.731 | 0.064 | 0.827 |
| STT | | | | | | | | | |
| STT-A | rEAP | **-0.488** | **0.034** | -0.138 | 0.572 | -0.094 | 0.701 | -0.097 | 0.691 |
| | HE | **-0.637** | **0.014** | -0.333 | 0.245 | -0.069 | 0.815 | 0.086 | 0.769 |
| STT-B | rEAP | -0.182 | 0.456 | -0.246 | 0.309 | 0.253 | 0.297 | 0.100 | 0.684 |
| | HE | 0.225 | 0.438 | 0.287 | 0.320 | -0.239 | 0.411 | 0.038 | 0.897 |
| SDMT | rEAP | 0.049 | 0.843 | 0.190 | 0.437 | -0.268 | 0.267 | -0.206 | 0.397 |
| | HE | 0.020 | 0.946 | 0.196 | 0.503 | -0.056 | 0.848 | 0.079 | 0.787 |

MoCA, Montreal Cognitive Assessment; MMSE, Mini-Mental State Examination; AVLT, Auditory Verbal Learning Test; VFT, Verbal Fluency Test; BNT, Boston Naming Test; STT, Shape Trail Test; SDMT, Symbol Digit Modalities Test; ADL, Activities of Daily Living Scale; Cingulum_Ant_R, right anterior cingulate/paracingulate cortex; Frontal_Sup_L, left dorsolateral superior frontal gyrus; RMTG, right middle temporal gyrus; LAG, left angular gyrus

**Supplementary Table 5.** Correlations between altered neuroimaging indexes and changes in neuropsychological test scores among aMCI subgroup

| Indicators | Group | ReHo (Frontal_Sup_L) | | ReHo (Cingulum_Ant_R) | | PCC-RMTG | | vmPFC-LAG | |
|---|---|---|---|---|---|---|---|---|---|
| | | **r** | ***p*** | **r** | ***p*** | **r** | ***p*** | **r** | ***p*** |
| MoCA | rEAP | 0.732 | 0.098 | 0.740 | 0.092 | -0.653 | 0.160 | -0.323 | 0.533 |
| | HE | 0.060 | 0.888 | 0.636 | 0.090 | -0.234 | 0.578 | -0.127 | 0.764 |
| MMSE | rEAP | **0.827** | **0.042** | **0.887** | **0.018** | -0.394 | 0.440 | -0.163 | 0.758 |
| | HE | **0.800** | **0.017** | 0.372 | 0.364 | -0.138 | 0.744 | 0.704 | 0.052 |
| AVLT | | | | | | | | | |
| Immediate-term | rEAP | -0.136 | 0.797 | -0.322 | 0.534 | 0.445 | 0.377 | 0.211 | 0.688 |
| | HE | 0.376 | 0.359 | -0.158 | 0.709 | 0.147 | 0.728 | 0.629 | 0.095 |
| Long-term | rEAP | -0.486 | 0.329 | -0.139 | 0.793 | -0.089 | 0.866 | -0.441 | 0.381 |
| | HE | 0.361 | 0.380 | 0.001 | 0.998 | -0.161 | 0.703 | 0.466 | 0.245 |
| Recall-term | rEAP | 0.456 | 0.364 | -0.229 | 0.662 | 0.123 | 0.817 | 0.352 | 0.494 |
| | HE | -0.189 | 0.654 | -0.370 | 0.367 | 0.461 | 0.251 | 0.373 | 0.363 |
| VFT | rEAP | 0.561 | 0.247 | 0.573 | 0.234 | 0.268 | 0.608 | 0.285 | 0.585 |
| | HE | 0.574 | 0.137 | 0.378 | 0.356 | -0.402 | 0.323 | -0.163 | 0.700 |
| BNT | rEAP | -0.372 | 0.468 | 0.076 | 0.886 | 0.219 | 0.677 | -0.051 | 0.923 |
| | HE | 0.435 | 0.281 | -0.118 | 0.782 | -0.151 | 0.722 | **0.727** | **0.041** |
| STT | | | | | | | | | |
| STT-A | rEAP | 0.036 | 0.945 | 0.570 | 0.238 | -0.091 | 0.864 | -0.232 | 0.658 |
| | HE | 0.696 | 0.055 | 0.071 | 0.867 | -0.120 | 0.778 | 0.481 | 0.227 |
| STT-B | rEAP | -0.261 | 0.617 | -0.019 | 0.972 | -0.743 | 0.090 | **-0.858** | **0.029** |
| | HE | 0.565 | 0.144 | 0.525 | 0.181 | 0.130 | 0.759 | 0.686 | 0.060 |
| SDMT | rEAP | 0.437 | 0.387 | 0.065 | 0.902 | 0.262 | 0.616 | 0.535 | 0.274 |
| | HE | **-0.808** | **0.015** | -0.094 | 0.824 | 0.091 | 0.830 | -0.227 | 0.589 |

MoCA, Montreal Cognitive Assessment; MMSE, Mini-Mental State Examination; AVLT, Auditory Verbal Learning Test; VFT, Verbal Fluency Test; BNT, Boston Naming Test; STT, Shape Trail Test; SDMT, Symbol Digit Modalities Test; ADL, Activities of Daily Living Scale; Cingulum_Ant_R, right anterior cingulate/paracingulate cortex; Frontal_Sup_L, left dorsolateral superior frontal gyrus; RMTG, right middle temporal gyrus; LAG, left angular gyrus
